# The impact of bisphosphonates on the osteoblast proliferation and Collagen gene expression *in vitro*

**DOI:** 10.1186/1746-160X-6-12

**Published:** 2010-07-09

**Authors:** Felix Peter Koch, Sareh Said Yekta, Christina Merkel, Thomas Ziebart, Ralf Smeets

**Affiliations:** 1 Department of Oral and Maxillofacial Surgery, University medical centre of the Johannes Gutenberg University Mainz, Augustusplatz 2, Mainz, Germany; 2 Department of Oral and Maxillofacial Surgery, University Hospital Aachen, Aachen, Germany; 3 Department of Operative Dentistry, Periodontology and Preventive Dentistry, University Hospital Aachen, Aachen, Germany

## Abstract

**Background:**

Bisphosphonates are widely used in the clinical treatment of bone diseases with increased bone resorption. In terms of side effects, they are known to be associated with osteonecrosis of the jaw (BONJ).

The objective of this study was to evaluate the effect of bisphosphonates on osteoblast proliferation by cell count and gene expression analysis of cyclin D1 *in vitro*. Furthermore, the gene expression of the extracellular matrix protein collagen type I was evaluated. Nitrogen-containing and non-nitrogen-containing bisphosphonates have been compared on gene expression levels.

**Methods:**

Human osteoblast obtained from hip bone were stimulated with zoledronate, ibandronate and clodronate at concentrations of 5 × 10^-5^M over the experimental periods of 1, 2, 5, 10 and 14 days. At each point in time, the cells were dissolved, the mRNA extracted, and the gene expression level of cyclin D1 and collagen type I were quantified by Real-Time RT-PCR. The gene expression was compared to an unstimulated osteoblast cell culture for control.

**Results:**

The proliferation appeared to have been influenced only to a small degree by bisphosphonates. Zolendronate led to a lower cyclin D1 gene expression after 10 days. The collagen gene expression was enhanced by nitrogen containing bisphosphonates, decreased however after day 10. The non-nitrogen-containing bisphosphonate clodronate, however, did not significantly influence cyclin D1 and collagen gene expression.

**Conclusions:**

The above data suggest a limited influence of bisphosphonates on osteoblast proliferation, except for zoledronate. The extracellular matrix production seems to be initially advanced and inhibited after 10 days. Interestingly, clodronate has little influence on osteoblast proliferation and extracellular matrix production in terms of cyclin D1 and collagen gene expression.

## Background

Bisphosphonates are widely used in the clinical treatment of bone diseases with increased bone resorption [1] such as Paget's disease, osteoporosis, and malignant diseases like multiple myeloma or metastasis to the bone. The increased bone mineral density has been attributed to a decreased bone turnover [2-5] by the inhibition of osteoclastic bone resorption.

There is, however, increasing evidence, that bisphosphonates interact with osteoblasts. The bisphosphonates are a family of pyrophosphate analogs that can further be separated into nitrogen-containing and non-nitrogen-containing bisphosphonates. Non-nitrogen-containing bisphosphonates are build into ATP resulting in a non-hydrolysable adenine containing metabolite, whereas nitrogen-containing bisphosphonates interfere with the mevalonate pathway by inhibition of farnesyl pyrophosphate (FPP) synthase enzyme [6,7]. This interference causes a reduction in geranyl geranyl diphosphate (GGPP), which is required for the prenylation of guanosin triphosphate (GTP)-binding proteins such as Rab, Rac, Ras, Rho and Cdc42 [8-12]. In contrast to older *in vivo *studies that attribute higher bone density to reduced bone turnover, newer studies have shown the potential of bisphosphonates to enhance osteoblast proliferation and differentiation in bone marrow-derived mesenchymal stem cells (MSC) and osteoblasts [13-15]. These actions could cause an altered cell metabolism, which is supposed to promote osteonecrosis that almost always occurs in the jaw as a serious side effect with exposed bone, fistulae and even pathological fractures [16,17]. Especially after treatment by nitrogen containing bisphosphonates intravenously an incidence of 5%-19% has been reported [18-20]. In addition to a direct effect on osteoclasts and osteoblasts, some authors suggest that a bisphosphonate induced obliteration of the regional blood vessels could lead to an avascular osteonecrosis of the jaw [17,21,22].

The objective of this *in vitro *study was to illuminate the impact of bisphosphonates on osteoblast proliferation and extracellular matrix production over a period of 14 days. Therefore, the genes of cyclin D1 and collagen were quantified by Real Time RT-PCR. The nitrogen-containing bisphosphonates zoledronate and ibandronate were compared to the non-nitrogen-containing bisphosphonate clodronate.

## Methods

### Cell culture

Human hip bone osteoblasts (HOB-c, Promo Cell, Heidelberg, Germany) between passages 5-9 were cultured at a density of 200 000 cells per well using 6-well plates. They were allowed to attach for two days using an osteoblast specific medium (10% FCS/DMEM Dulbecco modified medium (Invitrogen, Carlsbad, Ca/US) containing 1% L-glutamin, 1% penicillin/streptomycin/neomycin, 1% ascorbic acid, and 20 μg/ml dexamethasone. The cells were stimulated by osteoblast specific medium containing zoledronate, ibandronate, or clodronate at a concentration of 5 × 10^-5^M. The osteoblast specific cell culture medium without bisphosphonate supplement was used for control. The media and bisphosphonates were renewed every 4 days for a period of 14 days to guarantee a constant stimulation und nutrition supply over the experimental period.

### mRNA extraction and reverse transcriptase polymerase chain reaction (RT-PCR)

On day 1, 2, 5, 10, and 14 of cultivation, the osteoblasts were detached with 0.05% trypsin-EDTA solution (Invitrogen, Carlsbad, Ca, US) and individually harvested. MRNA was extracted using a silicate gel technique that was provided by the Qiagen RNeasy extraction kit (Qiagen, Hilden, Germany). This included a DNAse digestion step. The amount of extracted mRNA was measured by extinction at 260nm; the contamination with proteins was determinated with the 260/280 ratio.

To detect the mRNA of cyclin D1 and collagen type I in osteoblasts, primers were designed using NCBI-nucleotide library and Primer3-design (Tab. 1). All primers had been matched to the mRNA sequences of the target genes (NCBI Blast software).

As housekeeping genes, human ribosomal protein (HuPO), actin, glyceraldehyde-3-phosphate dehydrogenase (GAPDH) and ribosomal protein S18 (RPS18) were evaluated. We were able to show the most stable expression for the actin, GAPDH and RPS18 genes by comparing the bisphosphonate stimulated versus a non stimulated cell-culture using a specialized freeware, called GeNorm.

As a quantitative RT-PCR we used the SYBR Green Real Time PCR (oneStep RT-PCR, Bio-Rad, Hercules, CA/USA). This method enables reverse transcription using the individual primers immediately before PCR amplification and SYBR Green fluorescence measurement for quantification of gene expression. Samples were amplified in 96-well microplates in an IQ5-Cycler (Bio-Rad, Hercules, CA/USA) with an annealing temperature of 56°C and an elongation temperature of 71°C over 40 cycles. Background was to determine over 3-10 cycles and the threshold was set above this fluorescence, crossing the SYBR green fluorescence curve at the exponential part. This method was applied to calculate the cycle number and C_T_-value for quantitation. Furthermore, the C_T_-values of actin, GAPDH and RPS18 housekeeping genes and the individual primer efficacy were considered. Single product formation was confirmed by melting point analysis. For negative control, water instead of mRNA-samples was used.

CDNA from individual cell experiments was analyzed in triplicate PCR. The ΔΔC_T _method was applied [23,24] for a statistical analysis of the C_T_-values. For each specific primer and Real-Time PCR, the efficiency was calculated on the basis of the SYBR Green fluorescence curves and the standard dilution series. The relative gene expression levels were standardized with those measured in the unstimulated control, which was set to 100%. Each point in time for relative mRNA is the mean +/- standard deviation. (See Table 1)

**Table 1:** Oligonucleotide primer sequences used for Real Time PCR

	Sense	Antisense
**Cyclin D1**	ATCTCTGTACTTTGCTTGCT	AGTACATGGATATTCCCAAA

**Collagen I**	AGAACTGGTACATCAGCAAG	GAGTTTACAGGAAGCAGACA

**GAPDH**	AAAAACCTGCCAAATATGAT	CAGTGAGGGTCTCTCTCTTC

**RPS 18**	TCGGAACTGAGGCCATGA	GAACCTCCGACTTTCGTTC

### Statistical analysis

The mean values and standard deviations were calculated by the IQ5-software (BioRad, Hercules, CA/USA) to provide a descriptive data analysis.

## Results

### Effect of bisphosphonates on cyclin D1 gene expression

**Figure 1:**
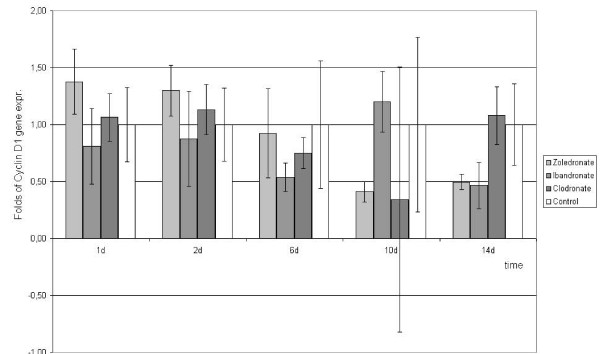
**Quantitative RT-PCR-results of cyclin D1 gene expression as fold of unstimulated control gene expression (means +/- SD), that was set at 1 (100%)**.

Time-course experiments were performed to determine the effects of zoledronate, ibandronate and clodronate on cyclin D1 gene expression. As shown in figure 1, treatment of human hip bone osteoblast [hOB] cells with ibandronate, zoledronate and clodronate did not significantly influence the gene expression of cyclin D1 during the first 6 days. Zoledronate, however, caused a decreasing cyclin D1 gene expression after the 6th day whereas ibandronate and clodronate did not significantly show enhanced or decreased gene expression levels.

### Effect of bisphosphonates on collagen gene expression

The collagen gene expression was stimulated to the most extent by ibandronate, reaching a maximum of 400% at day 10 compared to the non-stimulated control. Zoledronate also caused osteoblasts to increase their gene expression to a maximum level of 330% on day 2. After 14 days of stimulation the gene expression of collagen type I has decreased to a level of 12% for zoledronate, respectively 30% for ibandronate compared to an unstimulated control.

**Figure 2:**
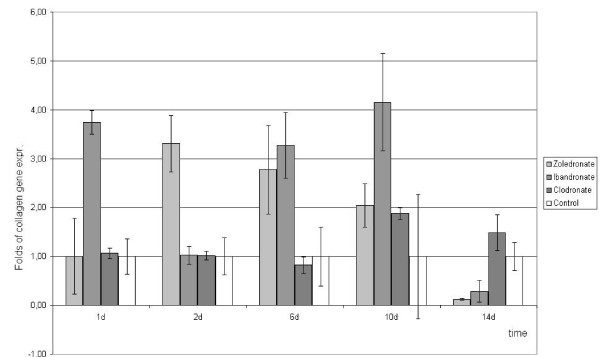
**Quantitative RT-PCR-results of type I collagen gene expression as fold of unstimulated control gene expression (means +/- SD), that was set at 1 (100%)**.

The non-nitrogen-containing clodronate, however, did not cause a significant alteration of collagen gene expression (figure 2).

## Discussion

Bisphosphonates are therapeutically applied to treat metabolic bone diseases, such as osteoporosis or metastasis to the bone. Clinical studies have shown their potency to increase bone density over an extended period of time [25-28]. This effect is not only caused by a positive bone turnover, but also by a direct stimulation of osteoblast and osteoblast precursor cells by applying nitrogen-containing bisphosphonates [15,29]. An anabolic effect to the bone could be caused by proliferation and by extracellular matrix production, mainly of collagen type I. With respect to osteoblast proliferation, we examined cyclin D1, an important regulator of the cell cycle and a surrogate of cell proliferation. Our results did not show a significant impact on osteoblast proliferation during the first 6 days. However, after day 6 zoledronate led to a reduced Cyclin D1 gene expression. As shown in other *in vitro *studies, pamidronate, a nitrogen-containing bisphosphonate, decreased osteoblast proliferation in a dose dependent manner [29]. In contrast, bisphosphonates have been reported to induce proliferation of marrow osteoprogenitors [30]. These anabolic effects are evidenced by a positive bone turnover, evaluated in clinical studies of up to 10 years of bisphosphonate treatment [25,31].

With respect to extracellular matrix production and early bone differentiation, type I collagen is the most important matrix protein. It is produced by osteoblasts and permits bone mineralization. The nitrogen-containing bisphosphonates appeared to induce collagen type I gene expression during the first 10 days. At day 14 the collagen gene expression level was lowered by the nitrogen containing bisphosphonates below 30%. These results are confirmed by Reinholz et al., who also found an enhanced collagen production [29]. The bisphosphonate as well effect bone marrow stromal cells by an enhanced collagen gene expression [15].

Our data suggest that nitrogen-containing bisphosphonate treatment enhances the differentiation of the osteoblasts from the proliferation stage into a nonproliferating matrix maturation stage. The lower proliferation but higher bone density through differentiation could explain the missing regeneration potential of BONJs.

In contrast, the non-nitrogen-containing bisphosphonate clodronate did not have any significant impact on osteoblasts cyclin D1 gene expression or type I collagen gene expression. These results support the assumption, that for the inhibition of farnesyl pyrophosphate (FPP) synthase enzyme [6,7] non-nitrogen-containing bisphosphonates mainly effect the osteoclasts, but not the osteoblasts. This was also confirmed by the clinically higher potency of nitrogen-containing bisphosphonates for bone density evaluation and a lower incidence of BONJ.

## Conclusions

Our data suggest that there is an antiproliferative effect of bisphosphonates on osteoblasts. Bisphosphonates, however, appear to enhance extracellular matrix production of collagen type I. The enhanced bone density mediated by bisphosphonates appears to be caused by the stimulation of osteoblast differentiation. Non-nitrogen-containing bisphosphonates do not appear to influence osteoblast proliferation and extracellular matrix production.

## Competing interests

The authors declare that they have no competing interests.

## Authors' contributions

FK conceived of the study, organized and carried out the PCR studies, designed the primers and drafted the manuscript. CK carried out the PCR studies as well. TZ participated in the design of the study. RS and SSY participated in the study design, supported by scientific consulting and coordination and helped to draft the manuscript. All authors read and approved the final manuscript.
